# Carotenoids Inhibit Fructose-Induced Inflammatory Response in Human Endothelial Cells and Monocytes

**DOI:** 10.1155/2020/5373562

**Published:** 2020-04-28

**Authors:** Ping Lin, Qian Ren, Qin Wang, Jiali Wu

**Affiliations:** Department of Geriatric, The 3rd Hospital of Hangzhou, Zhejiang 310009, China

## Abstract

**Objective:**

This research is aimed at determining the vascular health characteristics of carotenoids by evaluating their effect on excessive inflammatory response in endothelial and monocyte cells, the main factors of atherosclerosis.

**Methods:**

Human umbilical vein endothelial cells (HUVECs) or U937 monocytes were treated with escalating concentrations (0.1, 0.5, and 1 *μ*M) of five most common carotenoids in human plasma, i.e., *α*-carotene, *β*-carotene, *β*-cryptoxanthin, lutein, and lycopene prior to stimulation with 2 mM fructose. We examined the monocyte adhesion to endothelial cells (ECs) and relevant endothelial adhesion molecules. Chemokine and proinflammatory cytokine production as well as intracellular oxidative stress were also assessed in fructose-stimulated ECs and monocytes.

**Results:**

Carotenoids repressed monocyte adhesion to fructose-stimulated ECs dose dependently via decreasing primarily the expression of endothelial VCAM-1. In ECs and monocytes, three carotenoids, i.e., *β*-cryptoxanthin, lutein, and lycopene, suppressed the fructose-induced expression of chemokines MCP-1, M-CSF, and CXCL-10 and inflammatory cytokines TNF-*α* and IL-1*β*, with CXCL-10 being the most repressed inflammatory mediator. *β*-Cryptoxanthin, lutein, and lycopene dramatically downregulated the fructose-induced CXCL-10 expression in vascular cells. The reduction in the inflammatory response was associated with a slight but significant decrease of intracellular oxidative stress.

**Conclusions:**

Our results show that carotenoids have a variety of anti-inflammatory and antiatherosclerosis activities, which can help prevent or reduce fructose-induced inflammatory vascular diseases.

## 1. Introduction

Atherosclerosis is a chronic arterial wall inflammatory disease mediated by mast cells and innate/adaptive immune responses [1, 2]. It is the fundamental source of most cardiovascular diseases, which is the leading cause of death in the Western world today attributable to the high prevalence of metabolic risk factors, such as obesity and diabetes [3]. Atherosclerotic risk factors cause early activation and endothelial cell (EC) malfunction, accompanied by expression and release of adhesion molecules and chemokines, which lead to subintimal mononuclear leukocyte infiltration, the earliest morphological indicator of cardiovascular inflammation [4]. Once locally generated chemokines and cytokines enter the intima, they can irreversibly activate leukocytes, accelerate the transformation of monocytes into foam cells, and stimulate the expression of endothelial adhesion molecules such as E-selectin, vascular cell adhesion molecule-1 (VCAM-1), and intercellular adhesion molecule-1 (ICAM-1), thus promoting the recruitment, adhesion, and migration of leukocytes in inflammatory vascular walls [5, 6].

Dietary habits are essential and modifiable factors for chronic inflammatory conditions [7]. Fructose, a simple carbohydrate, exists as a free hexose in fruit and honey, and also as a free glucose-fructose mix in high-fructose corn syrup [8]. The primary source of fructose is sugar-sweetened drinks and processed foods. Fructose metabolism may lead to a depletion of ATP intracellular increase of uric acid production, oxidative stress, inflammation, and enhanced lipogenesis, which can trigger endothelial dysfunction and vascular wall damage [9]. There is growing concern about the possible role of sweeteners that contain fructose in the incidence of cardiovascular diseases (CVD) [10, 11]. A causal relationship was also suggested in recent years between the consumption of fructose and the worldwide obesity epidemic [12]. Excessive energy consumption from fructose was found to contribute to increased production of hepatic glucose, de novo lipogenesis, intrahepatic fat, hyperuricemia, and ultimate development of cardiovascular events [13, 14]. Importantly, fructose can cause endothelial dysfunction through a variety of pathways, including nitric oxide and endothelial nitric oxide synthase, endothelin-1, thromboxane a2, matrix metalloproteinase-2, and angiotensin II, eventually leading to cardiovascular damage [15]. Epidemiological studies have also found that increased consumption of sweetened drinks and fructose can enhance the risk of cardiac disease [16], high blood pressure, and metabolic syndrome [17]. However, there are some contradictions with respect to fructose-containing sugars and obesity, cardiovascular disease, and diabetes [18, 19], and the mechanism of cardiovascular inflammation caused by fructose is uncertain. Further studies have been proposed to explain current concerns about fructose toxicity [20].

Many natural molecules seem to have good effects on oxidative stress and vascular dysfunction [21, 22]; amongst them, carotenoids are the most clearly studied. Carotenoid is a huge family of fat-soluble molecules known for their antioxidant effects [23]. Furthermore, they also have an anti-inflammatory effect in addition to their antioxidant effect and play a critical role in preventing cardiovascular complications [24]. Out of over 700 carotenoids found, *β*-carotene and lycopene, which have free radical scavenger activity and nutritional significance, are the most well characterized [25]. Intriguingly, multiple studies have shown that carotenoids can prevent and alleviate diabetes and related complications through suppressing oxidative stress [26]. In preclinical studies, employing streptozotocin- (STZ-) induced diabetic rats as a model for evaluating the impact of chronic lycopene treatment [27–29], it was observed that this molecule functions as an antidiabetic agent, dampening endothelial dysfunction through its antioxidant effect. Other studies have also suggested that dietary lycopene application significantly decreased serum lipid levels and atherosclerotic plaque formation in New Zealand White rabbits fed a high-fat diet (HFD) [30], further suggesting that lycopene may play an important role in preventing cardiovascular events.

Considering carotenoids' favorable chemical features, we explored their possible anti-inflammatory pharmaceutical properties in this report. To this end, we employed cultured human ECs and monocyte stimulated by fructose, a proinflammatory and atherosclerotic factor [11], as models of vascular inflammation and atherosclerosis. By assessing the monocyte adhesion to inflamed ECs, the first imperative step of atherosclerosis progression [31], and the relevant endothelial expression of adhesion molecules, we examined the anti-inflammatory activities of some major components from carotenoids. In addition, we also examined the gene expression of inflammation mediators, such as chemokines and proinflammatory cytokines in fructose-activated ECs and monocytes.

## 2. Materials and Methods

### 2.1. Materials

The cell culture materials were purchased from Invitrogen (Carlsbad, CA, USA). CXCL-10 assay kit, antibody against VCAM-1, and peroxidase-conjugated secondary antibody were from Abcam. All other reagents were obtained from Sigma-Aldrich, unless otherwise stated. *β*-Cryptoxanthin was obtained from Funakoshi Co., Ltd. (Tokyo, Japan).

### 2.2. Cell Culture and Treatment

Human umbilical vein endothelial cells (HUVECs) were purchased from Gibco and maintained in medium 200 supplemented with low serum growth supplement (LSGS), 95% humidified air, and 5% CO_2_ at 37°C. The human U937 cells were obtained from the ATCC (Rockville, MD) and cultured in RPMI medium 1640 containing 10% fetal bovine serum (FBS) (Biological Industries), 100 U/ml penicillin, and 100 *μ*g/ml streptomycin. In order to avoid cell differentiation, U937 cell density was maintained below 1 × 10^6^ cells/ml. For treatment, confluent ECs or U937 cells were moved to medium containing 3% FBS for 4 h and then treated with or without *β*-cryptoxanthin, lutein, and lycopene at various concentrations (0.1, 0.5, and 1 *μ*M), followed by stimulation with fructose 2 mM for further 4–16 h. Cell toxicity was tested with Trypan blue exclusion assays.

### 2.3. Monocyte Endothelium Adhesion Assay

ECs were cultured to confluence in 6-well tissue culture plates, treated with or without *β*-cryptoxanthin, lutein, and lycopene (0.1, 0.5, and 1 *μ*M) for 2 h and further incubated with fructose 2 mM for 16 h. U937 cells were labeled with calcein AM (1 *μ*M) for 40 min in medium containing 3% FBS. For the coculture system, the labeled U937 cells (5 × 10^5^) were seeded on EC monolayer, as previously described [32]. After washing, five photomicrographs were obtained from each well and counted with an NHI Image Analyzer system under a fluorescence microscope. Alternately, each well's fluorescence intensity was determined by using a microplate reader (485/530 nm wavelength).

### 2.4. EC Surface Molecule Assay

HUVECs were cultured to confluence and treated with or without *β*-cryptoxanthin, lutein, and lycopene at various concentrations (0.1, 0.5, and 1 *μ*M) for 2 h, followed by stimulation with 2 mM fructose for 6 h for E-selectin or 16 h for ICAM-1/VCAM-1. Assays of cell surface molecules were performed by using enzyme immunoassays (EIA) with primary anti-human antibodies against VCAM-1, ICAM-1, and E-selectin or antibody against noncytokine-inducible and constitutive EC antigen E1/1, as described previously [33].

### 2.5. Chemokine Release

ECs or U937 cells were incubated with *β*-cryptoxanthin, lutein, and lycopene for 2 h and then stimulated with 2 mM fructose for 24 h. The conditioned media were used to measure the secreted CXCL-10 by using an ELISA kit according to the manufacturer's instructions.

### 2.6. Quantitative RT-PCR Analysis

ECs or U937 cells were pretreated with *β*-cryptoxanthin, lutein, and lycopene for 2 h, followed by stimulation with 2 mM fructose for 4 h. Total RNA was extracted using the TRIzol reagent (Invitrogen) in keeping with the manufacturer's protocol. A cDNA Reverse Transcription Kit (Applied Biosystems) was used for converting the total RNA (2 *μ*g) into the first-strand cDNA. The qRT-PCR was carried out with the SYBR Green PCR Master Mix in the Biorad CFX384 Real-Time PCR Detection System. The cDNA fragments were amplified with the primers shown in Table 1. GAPDH mRNA were used as the internal control.

### 2.7. Lipid Peroxidation

The cellular lipid peroxidation amount was assessed by the generation of thiobarbituric acid reactive species (TBARS) according to the Esterbauer and Cheeseman method [34, 35]. In brief, malondialdehyde (MDA), a lipid peroxidation by-product, forms an adduct with thiobarbituric acid (TBA), which can be colorimetrically determined by the MDA equivalent standard. Each sample was added with butylated hydroxytoluene to avoid further lipid oxidation throughout sample processing as well as the TBA reaction. The production of MDA, indicated as nanomoles/mg protein, was spectrometrically monitored at 533 nm.

### 2.8. Intracellular ROS Determination

Intracellular ROS production was detected using a DCFH-DA in fructose or carotenoid-treated HUVECs. HUVECs were pretreated with *β*-cryptoxanthin, lutein, and lycopene (1 *μ*M) or vehicle (control) for 2 h and then incubated with 2 mM fructose for 16 h. The cells were incubated with 20 *μ*M DCFH-DA (final concentration) in PBS for 20 min at 37°C. Following washing with PBS, a fluorescence microscopy was used to monitor the production of the intracellular ROS and a GENios microplate reader was employed to quantify the intensity of DCF fluorescence at the wavelength Ex/Em 485 nm/535 nm.

### 2.9. Statistical Analysis

Results of at least 3 independent tests are expressed as the mean ± SD. Unpaid Student's *t*-tests were used to determine the differences between two groups. The one-way ANOVA was used for multiple comparisons, and after the presentation of substantial intergroup difference by ANOVA, individual differences were then checked with Fisher's protected least significant difference test. A *p* value < 0.05 was statistically significant.

## 3. Results

### 3.1. Carotenoids Inhibit Monocyte Adhesion to Fructose-Stimulated Endothelial Cells and Suppress Endothelial Adhesion Molecule Expression

**Figure 1:**
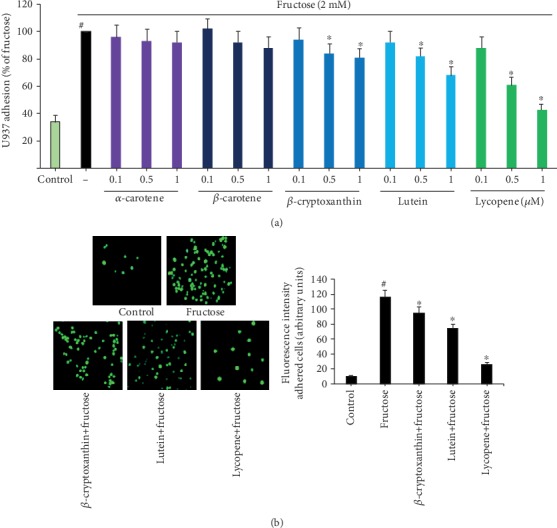
Inhibitory effects of carotenoids on adhesion of monocytes to fructose-stimulated endothelial cells. HUVECs were pretreated with the five most common carotenoids in human plasma, i.e., *α*-carotene, *β*-carotene, *β*-cryptoxanthin, lutein, and lycopene (0.1, 0.5, and 1 *μ*M) or vehicle (control) for 2 h and then stimulated with fructose 2 mM for 16 h. Then, cells were cocultured with U937 monocytes labeled with calcein AM. The adherent quantity of U937 cells was monitored by a fluorescence plate reader (a) or fluorescence microscope (b). The adhesion values of monocytes are represented in comparison to monocyte adhesion to fructose-stimulated ECs, normalized at 100% (a). (b) Representative images of EC-monocyte adhesion after preincubation with *β*-cryptoxanthin, lutein, and lycopene (1 *μ*M). Each experiment was conducted in triplicate. ^#^*p* < 0.01 vs. control; ^∗^*p* < 0.05 vs. fructose alone.

To assess the effects of carotenoids on endothelial cell-monocyte adhesion, a critical step in an inflammatory and atherosclerotic process, HUVECs were preexposed to various carotenoids *α*-carotene, *β*-carotene, *β*-cryptoxanthin, lutein, or lycopene at increasing concentrations (0.1, 0.5, and 1 *μ*M), before stimulation with fructose (2 mM). Monocytoid cells do not adhere to unstimulated EC monolayers (control) but strongly adhered to fructose-stimulated ECs (Figure 1). The treatment of ECs with *β*-cryptoxanthin, lutein, or lycopene decreases fructose-induced monocyte adhesion in a dose-dependent manner (Figure 1), with an inhibitory effect already obvious at 0.5 *μ*M. As shown in the bar graph (Figure 1(a)) and representative images (Figure 1(b)), *β*-cryptoxanthin, lutein, or lycopene have the greatest inhibitory effect on monocyte adhesion to fructose-treated ECs at a concentration of 1 *μ*M (about 19%, 32%, or 57%, respectively). It is worth noting that the inhibitory effect of carotenoids only occurs under the proinflammatory conditions simulated by fructose stimulation, without impacting the expression of the constitutive endothelial surface antigen E1/1 (data not shown). The treatment with carotenoids does not considerably change the EC viability, as detected by MTT assay. Trypan blue assay also substantiated that the cell viability is higher than 95% under all conditions (data not shown).

**Figure 2:**
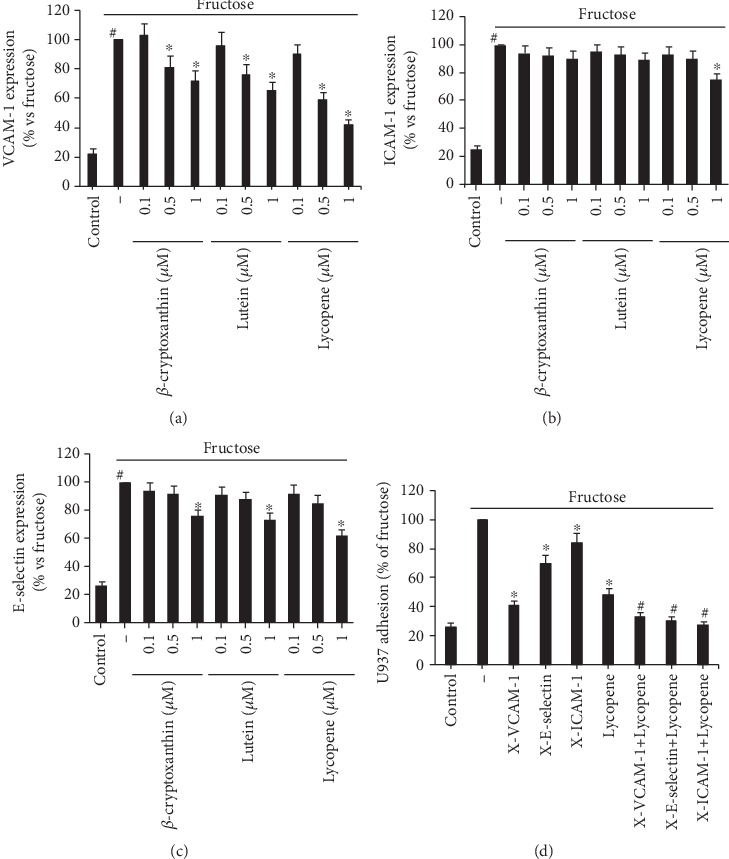
Dose-dependent effect of carotenoids on the expression of endothelial adhesion molecules. ECs were preincubated with *β*-cryptoxanthin, lutein, and lycopene (0.1, 0.5, and 1 *μ*M) or vehicle (control) for 2 h and then treated with fructose 2 mM. VCAM-1 (a), ICAM-1 (b), and E-selectin (c) expression levels were examined with cell surface EIA. Each experiment was conducted three times. Data are shown as the percentage of expression induced by fructose (mean ± SD). ^#^*p* < 0.01 vs. control; ^∗^*p* < 0.05; ^∗∗^*p* < 0.01 vs. fructose alone. (d) During the last 30 minutes of HUVEC stimulation with fructose (2 mM, 16 h) in the presence or absence of lycopene (1 *μ*M) and just before the monocyte adhesion test, neutralizing anti-VCAM-1, anti-E-selectin, or anti-ICAM-1 antibodies (5 *μ*g/ml for all) were introduced. This leads to inhibition of fructose-induced adhesion (*n* = 3). ^∗^*p* < 0.05 vs. fructose-stimulated control cells; ^#^*p* < 0.05 vs. fructose/lycopene.

Given the fact that monocytes binding to endothelium are regulated by the elevated expression of endothelial adhesion molecules, we analyzed the influence of carotenoids on the expression of VCAM-1, ICAM-1, and E-selectin with cell surface EIA. The expression of endothelial adhesion molecules was found in unstimulated HUVECs at relatively low levels, and it increased considerably following fructose treatment (Figure 2). Three carotenoids influenced the fructose-induced expression of endothelial adhesion molecules, particularly VCAM-1. As illustrated in Figure 2(a), VCAM-1 expression was diminished by *β*-cryptoxanthin, lutein, or lycopene dose dependently, with obvious effects already noticeable at 0.5 *μ*M. Lycopene at concentration of 1 *μ*M significantly reduced fructose-induced ICAM-1 expression (Figure 2(b)). The expression of E-selectin was also dramatically reduced by three carotenoids only at a concentration of 1 *μ*M (Figure 2(c)). Utilizing neutralizing antibodies against human ICAM-1, VCAM-1, and E-selectin, we examined their specific involvement in fructose-induced monocyte adhesion to HUVECs (Figure 2(d)). While anti-ICAM-1 antibody produced modest (16 ± 5%), but significant, reductions in fructose-induced monocyte adhesion, anti-VCAM-1 and anti-E-selectin antibodies individually hindered the number of adherent monocytes by 59 ± 11% and 30 ± 14%, respectively. Lycopene can further increase the inhibitory effect of these three antibodies on monocyte adhesion, reinforcing the notion that carotenoids act by blocking these adhesion molecules.

**Figure 3:**
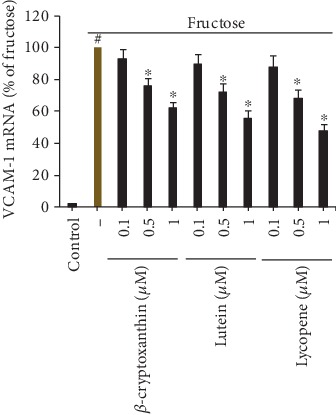
Effects of carotenoids in endothelial cells on fructose-stimulated VCAM-1 mRNA expression. HUVECs were pretreated with 0.1, 0.5, and 1 *μ*M carotenoids for 2 h before 2 mM fructose stimulation for a further 4 h; then VCAM-1 mRNA levels were measured with the quantitative RT-PCR. Results represent four independent studies (mean ± SD) as a percentage of fructose-stimulated ECs. ^#^*p* < 0.05 vs. control; ^∗^*p* < 0.05 vs. fructose alone.

In order to explore the possible mechanisms of the observed inhibitory effects on monocyte adhesion, we examined the endothelial expression of the gene coding VCAM-1, which is the primary endothelial adhesion molecule reduced by carotenoids. Quantitative real-time PCR analysis showed that carotenoids such as *β*-cryptoxanthin, lutein, and lycopene dose dependently decreased mRNA levels of VCAM-1 in fructose-stimulated ECs (Figure 3), thereby indicating an inhibitory effect of carotenoids at a pretranslation level.

### 3.2. Carotenoids Inhibit Inflammatory Response in Endothelial Cells

**Figure 4:**
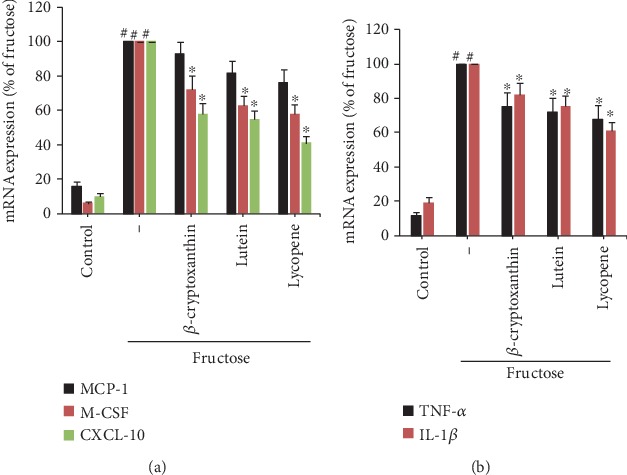
Effects of carotenoids on inflammatory marker expression in endothelial cells. ECs were pretreated for 2 h with *β*-cryptoxanthin, lutein, and lycopene (0.1, 0.5, and 1 *μ*M) or vehicle (control) and stimulated for 4 h with 2 mM fructose. Quantitative RT-PCR was used to evaluate the mRNA levels of MCP-1, M-CSF, and CXCL-10 (a) and TNF-*α* and IL-1*β* (b). The results are representatives of 3 fully independent experiments (mean ± SD), each conducted three times as a percentage of ECs stimulated with fructose. ^#^*p* < 0.05 vs. control; ^∗^*p* < 0.05 vs. fructose alone.

To enhance the understanding of carotenoids' vascular anti-inflammatory effects, we examined gene expression of EC inflammatory mediators namely chemokines and proinflammatory cytokines. Fructose treatment triggered a robust inflammatory response in ECs, elevating the mRNA expression of chemokines, such as monocyte chemoattractant protein-1 (MCP-1), macrophage colony-stimulating factor (M-CSF), and C-X-C motif ligand 10 (CXCL-10, also referred to as interferon-inducible protein 10 (IP-10)) (Figure 4(a)), and enhancing the proinflammatory cytokines including tumor necrosis of (TNF-*α*) and interleukin-1*β* (IL-1*β*) (Figure 4(b)). In fructose-stimulated ECs, three carotenoid components decreased the mRNA expressions of chemokines and cytokines, albeit in varying degrees (Figure 4). Chemokine CXCL-10 has been the most dramatically modulated by carotenoids amongst endothelial inflammatory mediators. In fact, all components have substantially suppressed more than 40% of the fructose-induced expression of CXCL-10. Similarly, the induced mRNA levels of M-CSF (Figure 4(a)), and TNF-*α*, and IL-1*β* (Figure 4(b)) were reduced considerably while the repression of the MCP-1 mRNA level was not significant (Figure 4(a)).

### 3.3. Carotenoids Alleviate the Highly Inflammatory Reaction in Fructose-Stimulated Monocytes

**Figure 5:**
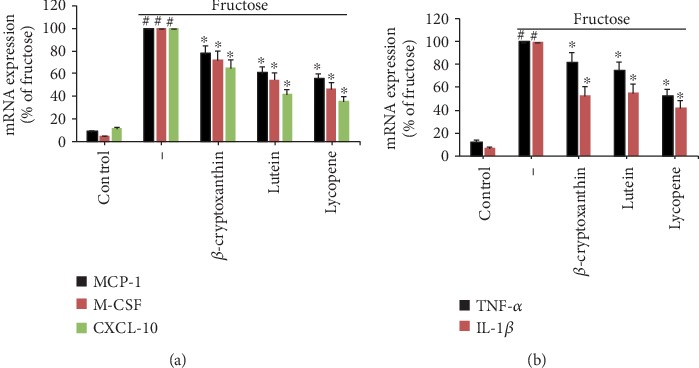
Effects of carotenoids on the expression of inflammatory markers in monocytes. U937 cells were pretreated with *β*-cryptoxanthin, lutein, and lycopene (0.1, 0.5, and 1 *μ*M) or vehicle (control) for 2 h and then incubated for 4 h with 2 mM fructose. Quantitative RT-PCR was used to assess the mRNA levels of MCP-1, M-CSF, and CXCL-10 (a) and TNF-*α* and IL-1*β* (b). The results are representatives of 3 fully independent experiments (mean ± SD), each conducted three times as a percentage of ECs stimulated with fructose. ^#^*p* < 0.05 vs. control; ^∗^*p* < 0.05 vs. fructose alone.

In order to evaluate the effects of carotenoids' anti-inflammatory activity in vascular cells other than endothelium, we explored the effects of three carotenoid components on fructose-activated human monocytic U937 cells and assessed the mRNA expression of chemokines and proinflammatory cytokines. Fructose stimulation significantly enhanced the gene expression of the MCP-1, M-CSF, CXCL-10, TNF-*α*, and IL-1*β* in monocytes (Figure 5). Overexpression of all studied chemokines and proinflammatory cytokines has been substantially blunted by *β*-cryptoxanthin, lutein, and lycopene in monocytes, with a higher inhibition effect than endothelium. Like ECs, CXCL-10 was also the primarily decreased inflammatory mediator by carotenoids in monocytes, achieving reduction by approximately 65%, 42%, or 39%, respectively, for *β*-cryptoxanthin, lutein, and lycopene (Figure 5). The anti-inflammatory activity of carotenoids was not correlated with their cytotoxic effects in monocytes as they did not substantially alter the cell viability as assessed by Trypan blue assay (data not shown).

### 3.4. Carotenoids Impede Release of Inflammation-Related Cytokines/Chemokines in Inflamed Endothelial and Monocytic Cells

**Figure 6:**
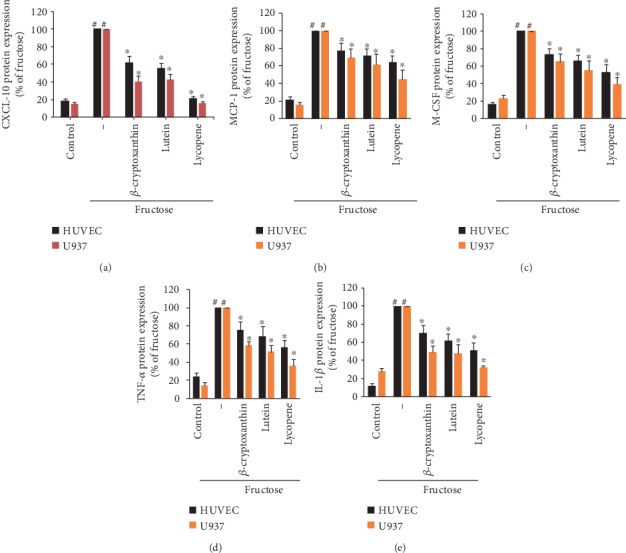
Inhibitory effects of carotenoids on the production of CXCL-10 protein in HUVECs or U937 monocytic cells induced by fructose. ECs or U937 cells were pretreated with *β*-cryptoxanthin, lutein, and lycopene (0.1, 0.5, and 1 *μ*M) or vehicle (control) for 2 h and then incubated with 2 mM fructose for 18 h. The protein production of CXCL-10 (a), MCP-1 (b), M-CSF (c), TNF-*α* (d), and IL-1*β* (e) was measured by ELISA in culture medium. Results are shown as the percentage of fructose-induced expression (mean ± SD) (*n* = 3). ^#^*p* < 0.05 vs. control; ^∗^*p* < 0.05 vs. fructose alone.

Because carotenoids significantly reduced mRNA expression of CXCL-10, MCP-1, M-CSF, TNF-*α*, and IL-1*β*, their inhibitory effect was confirmed in ECs and monocytes at the protein level. This inflammation-related cytokine/chemokine release in ECs and U937 cells was robustly induced by fructose stimulation versus unstimulated controls (Figure 6). Carotenoids significantly reduced the secretion of these cytokines/chemokines in fructose-stimulated ECs, but its effect on CXCL-10 was the most obvious, almost reducing to the level of nonstimulated control. Similar results were observed in U937 cells, where all analyzed carotenoid components significantly reduced CXCL-10 protein release.

### 3.5. Carotenoids Suppressed Oxidative Stress in Fructose-Stimulated Vascular Cells

**Figure 7:**
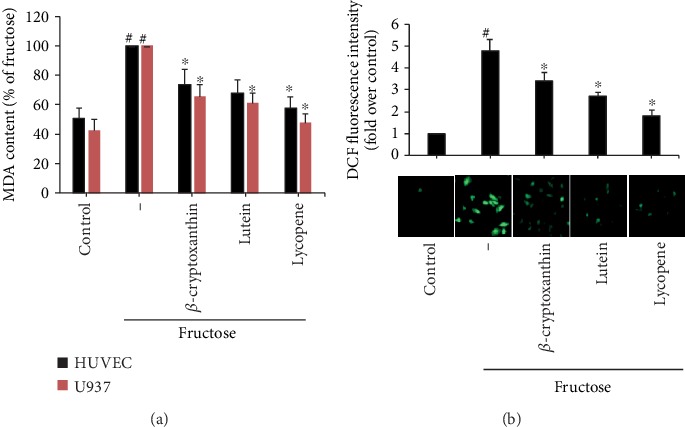
Effects of *β*-cryptoxanthin, lutein, and lycopene on fructose-induced oxidative stress in ECs and monocytes. HUVECs or U937 cells were pretreated with *β*-cryptoxanthin, lutein, and lycopene (1 *μ*M) or vehicle (control) for 2 h and then incubated with 2 mM fructose for 16 h. Lipid peroxidation was assessed by MDA content (a). (b) Determination of the intracellular ROS generation via DCFH-DA staining in fructose or carotenoid-treated HUVECs with a fluorescence microscope (lower panel) and quantitative intracellular DCF fluorescence (upper panel). The results are representatives of 3 fully independent experiments (mean ± SD) and indicated as the percentage of fructose-stimulated cells. ^#^*p* < 0.05 vs. control; ^∗^*p* < 0.05 vs. fructose alone.

It has been well known that inflammatory response triggered by fructose occurred through the excess production of intracellular reactive oxygen species (ROS) [36–38], which might elicit direct damage to cellular lipids, known as lipid peroxidation [39]. Here, we sought to determine the effects of carotenoids on fructose-triggered lipid peroxidation in ECs and monocytes, as indicated by their end product MDA levels [39].  Figure 7(a) illustrates that the stimulation of fructose generated elevated lipid peroxidation in HUVECs and U937 cells, as measured by MDA content. After exposure to *β*-cryptoxanthin, lutein, or lycopene, the MDA levels triggered by fructose stimulation reduced dramatically. The inhibitory effect of *β*-cryptoxanthin, lutein, or lycopene on intracellular ROS generation was further substantiated using DCFH-DA assay (Figure 7(b)).

## 4. Discussion

Some major epidemiological studies have demonstrated a link between increased plasma carotenoid levels and reduced risk of cardiovascular disorders [40]. In the present study, we investigated the anti-inflammatory effects of the major carotenoids (*α*-carotene, *β*-carotene, *β*-cryptoxanthin, lutein, or lycopene) by using an in vitro inflammatory and atherogenic model.

We observed that three important components from carotenoids (*β*-cryptoxanthin, lutein, and lycopene) dose dependently repressed monocyte adhesion to inflammatory endothelium. The first stage of atherosclerosis development is the mobilization of monocytes from circulation, their adherence to ECs, and resulting transendothelial migration to the intima of vascular walls [31]. Cell adhesion molecules including E-selectin, VCAM-1, and ICAM-1 on the activated endothelial surface participate in these interactions between monocytes and ECs [41]. Carotenoids blocked endothelial-monocyte adhesion through suppressing the expression of adhesion molecules, especially VCAM-1, which recognizes and attaches the VLA-4 counter-receptor to monocytes or lymphocytes, triggering adhesion at the activation site [42]. Leukocytes move to the underlying intima once they adhere to the endothelium under the stimulation of chemokines. Many types of chemokines may be involved in the recruitment of different classes of leukocytes for atherosclerotic lesions, including MCP-1, which attracts leukocytes carrying chemokine receptor CCR2 and the monokine CXCL-10 [43]. The M-CSF growth factor also facilitates the movement of monocytes into the vascular wall and prompts EC-derived MCP-1. We demonstrated that in fructose-triggered ECs, carotenoids decreased the gene expression of chemotactic cytokines such as M-CSF and CXCL-10. More pronounced inhibitory effects on the overwhelmingly inflammatory response of monocytes were observed, where carotenoids dramatically decreased mRNA of M-CSF, CXCL-10, and MCP-1, thus demonstrating multiple intervention to combat recruitment of leukocytes in the vascular wall. Other crucial proinflammatory cytokines such as TNF-*α* and IL-1*β* may stimulate leukocyte adherence and migration, which may worsen endothelial dysfunction [44]. Carotenoids reduced TNF-*α* and IL-1*β* gene expression in vascular cells, thereby intensifying the anti-inflammatory response.

Here, we showed new evidence of anti-inflammatory actions of carotenoids that were able to decrease the expression and release of CXCL-10 induced by fructose in activated endothelium/monocytes. CXCL-10 is believed to play a crucial role in recruiting activated T-cells in sites of tissue inflammation in many inflammatory diseases [45]. In addition, CXCL-10 is usually highly expressed in human atheroma during all stages of plaque formation, and serum CXCL-10 levels are correlated with coronary artery disease severity and occlusion of coronary arteries [46]. It is well known that oxidative stress mediates the proinflammatory response caused by fructose [11]. We demonstrated that carotenoids have anti-inflammatory effects associated with decreased intracellular oxidative stress, consistent with reduced lipid peroxidation levels. Together, our findings suggest that carotenoids exhibit antioxidant activity in fructose-stimulated vascular cells, confirming and expanding their antioxidant characteristics in acellular systems. Consistent with our results, a recent study [47] also reported anti-inflammatory roles of carotenoids (*β*-carotene and lycopene) in endothelial cells derived from the umbilical cord of women affected by gestational diabetes mellitus. In particular, pretreatment of *β*-carotene and lycopene significantly decreased the amount of peroxynitrite, leading to the protection of redox balance. The results indicate a new mechanism for the function of carotenoids that exert vascular defense in diabetic conditions, highlighting the value of a diet rich in carotenoids to avoid cardiovascular complications.

**Figure 8:**
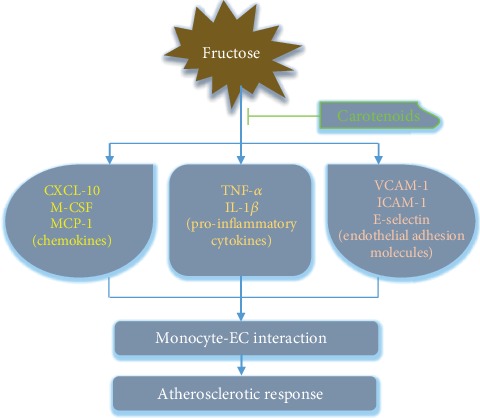
Schematic diagram about the anti-inflammatory properties of carotenoids in fructose-stimulated ECs and monocytes.

Overall, we suggest that *β*-cryptoxanthin, lutein, or lycopene is the three important components as functional carotenoids that can improve vascular function and provide health protection. They hinder endothelium-monocyte interaction and EC activation by downregulating adhesion molecules, chemoattractants, and inflammatory cytokines (Figure 8). In fructose-stimulated monocytes, these last two inflammatory mediators are also reduced, highlighting the role of carotenoids in resisting leukocyte activation, which is an imperative phenomenon in the formation and development of atherosclerosis as well as chronic inflammatory pathology.

In conclusion, our results show several biological health effects of carotenoids by regulation of gene expression implicated in monocyte adhesion, transendothelial migration, and immune response. These anti-inflammatory characteristics of carotenoids can help to mitigate or prevent inflammatory cardiovascular disorders.

## Figures and Tables

**Table 1:** Primer sequences of real-time quantitative PCR.

Gene	Accession number	Primers (sequence 5′–3′)	Size (bp)
TNF-*α*	NM_000594.2	AGGACACCATGAGCACTGAA CCGATCACTCCAAAGTGCAG	178
IL-1*β*	NM_000576.2	CTCTCTCCTTTCAGGGCCAA GCGGTTGCTCATCAGAATGT	154
VCAM-1	NM_00107B.3	TCAGATTGGAGACTCAGTCATGT ACTCCTCACCTTCCCGCTC	140
ICAM-1	NM_000201.2	GGCCGGCCAGCTTATACAC TAGACACTTGAGCTCGGGCA	190
E-selectin	NM_000450.2	AATGTGTGGGTCTGGGTAGG TCTTCTTGCTGCACCTCTCA	166
MCP-1	*NM_002982.3*	GCTCAGCCAGATGCAATCAA ACAGATCTCCTTGGCCACAA	159
M-CSF	NM_000757.4	CCCAGTGTCATCCTGGTCTT GTTCTGTGCGTCCAGCTTAG	187
CXCL-10	NM_001565.2	TGGATGTTCTGACCCTGCTT GGCAGTGGAAGTCCATGAAG	175
GAPDH	NG_007073.2	GACCATAGCAGGGACAAGGT TTCCTCCATCCCTGTTGTCC	197

## Data Availability

The data used to support the findings of this study are available from the corresponding author upon request.
